# Human Empathy Through the Lens of Social Neuroscience

**DOI:** 10.1100/tsw.2006.221

**Published:** 2006-09-20

**Authors:** Jean Decety, Claus Lamm

**Affiliations:** Department of Psychology Center for Cognitive and Social Neuroscience, University of Chicago, Chicago, IL 60637, USA

**Keywords:** empathy, intersubjectivity, affective sharing, perspective taking, emotion regulation, social neuroscience

## Abstract

Empathy is the ability to experience and understand what others feel without confusion between oneself and others. Knowing what someone else is feeling plays a fundamental role in interpersonal interactions. In this paper, we articulate evidence from social psychology and cognitive neuroscience, and argue that empathy involves both emotion sharing (bottom-up information processing) and executive control to regulate and modulate this experience (top-down information processing), underpinned by specific and interacting neural systems. Furthermore, awareness of a distinction between the experiences of the self and others constitutes a crucial aspect of empathy. We discuss data from recent behavioral and functional neuroimaging studies with an emphasis on the perception of pain in others, and highlight the role of different neural mechanisms that underpin the experience of empathy, including emotion sharing, perspective taking, and emotion regulation.

## INTRODUCTION

Among the various forms of emotional connections with others, empathy has received much attention from philosophers and psychologists, and more recently from cognitive neuroscientists. “Empathy” denotes, at a phenomenological level of description, a sense of similarity between the feelings one experiences and those expressed by others[1]. It can be conceived of as an interaction between any two individuals, with one experiencing and sharing the feeling of the other. Yet, the sharing of feelings is not sufficient to elicit empathy. Many scholars view empathy as an other-oriented social emotion (see Table 1). Moreover, the social and emotional situations that elicit empathy can become quite complex depending on the feelings experienced by the observer and the relationship of the target to the observer. In addition, numerous studies have documented that empathy plays a central role in moral reasoning, motivates prosocial behaviors, and inhibits aggression toward others (e.g., [2]). Batson and colleagues[3] offered an empathy-altruism hypothesis, which claims that purely altruistic action can reliably occur provided that it is preceded by empathic concern for another. Empathic concern is defined as an emotional reaction characterized by such feelings as compassion, tenderness, softheartedness, and sympathy.

**TABLE 1**
**Various Definitions of Empathy***

- The ability to put oneself into the mental shoes of another person to understand his or her emotions and feelings[*a form of simulation, or inner imitation*][4]
- A complex form of psychological inference in which observation, memory, knowledge, and reasoning are combined to yield insights into the thoughts and feelings of others[5]
- An affective response more appropriate to someone else's situation than to one's own[6]
- An other-oriented emotional response congruent with the other's perceived welfare[7]
- An affective response that stems from the apprehension or comprehension of another's emotional state or condition, and which is similar to what the other person is feeling or would be expected to feel in the given situation[8]

* Note that these definitions point to an emotional experience that is more congruent with another's situation than with one's own. Another important aspect of the construct of empathy is that it must involve some self-other differentiation, which makes it distinct from related reactions such as emotional contagion and personal distress.

The goal of this paper is to articulate different levels of analysis of the construct of empathy, including evolutionary, cognitive neuroscience, and social psychological descriptions. We believe that bridging these levels – the task of social neuroscience – can achieve a better understanding of such a complex aspect of human behavior. Bridging social psychology and cognitive neuroscience provides important guidelines for investigating the neural processes underlying empathy. Furthermore, cognitive neuroscience may help to disambiguate competing social psychology theories; for instance, concerning the effects of perspective taking on empathy and personal distress. To that end, instead of addressing each of these research domains separately, theoretical notions and findings from these different approaches will be marshaled with the guidance of an overarching conceptual framework (see Fig. 1).

This framework considers empathy as a construct that accounts for a sense of similarity in the feelings experienced by self and other (such translations go both ways, from other-to-self and from self-to-other), without confusion between the two agents. The experience of empathy often (but not always) results in sympathy (concern for another based on the apprehension or comprehension of the other's emotional state or condition), although it also can lead to empathic overarousal (or personal distress, an aversive, self-focused emotional reaction to the apprehension or comprehension of another's emotional state or condition). Our model of empathy involves both bottom-up and top-down information processing. Furthermore, it combines representational aspects, i.e., memories that are localized in distributed neural networks that encode information and, when temporarily activated, enable access to this stored information, as well as processes, i.e., computational procedures that are localized and are independent of the nature or modality of the stimulus that is being processed.

## EVOLUTIONARY ROOTS OF EMPATHY

Preston and de Waal[9] convincingly argued that empathy is not an all-or-nothing phenomenon, and many intermediate forms of empathy exist between the extremes of mere agitation at the distress of another and full understanding of their predicament. Other comparative psychologists view empathy as a kind of induction process by which emotions, both positive and negative, are shared, and by which the probabilities of similar behaviors are increased in the observers. While certain nonhuman primates may share feelings between individuals, humans are uniquely able to intentionally feel for and act on behalf of other people whose experiences may differ greatly from their own[3]. Such a capacity may help to explain why empathic concern is often associated with prosocial behaviors such as helping a kin, and has been considered crucial for altruism. Evolutionary biologists suggest that empathic helping behavior has evolved because of its contribution to genetic fitness (kin selection). In humans and other mammals, an

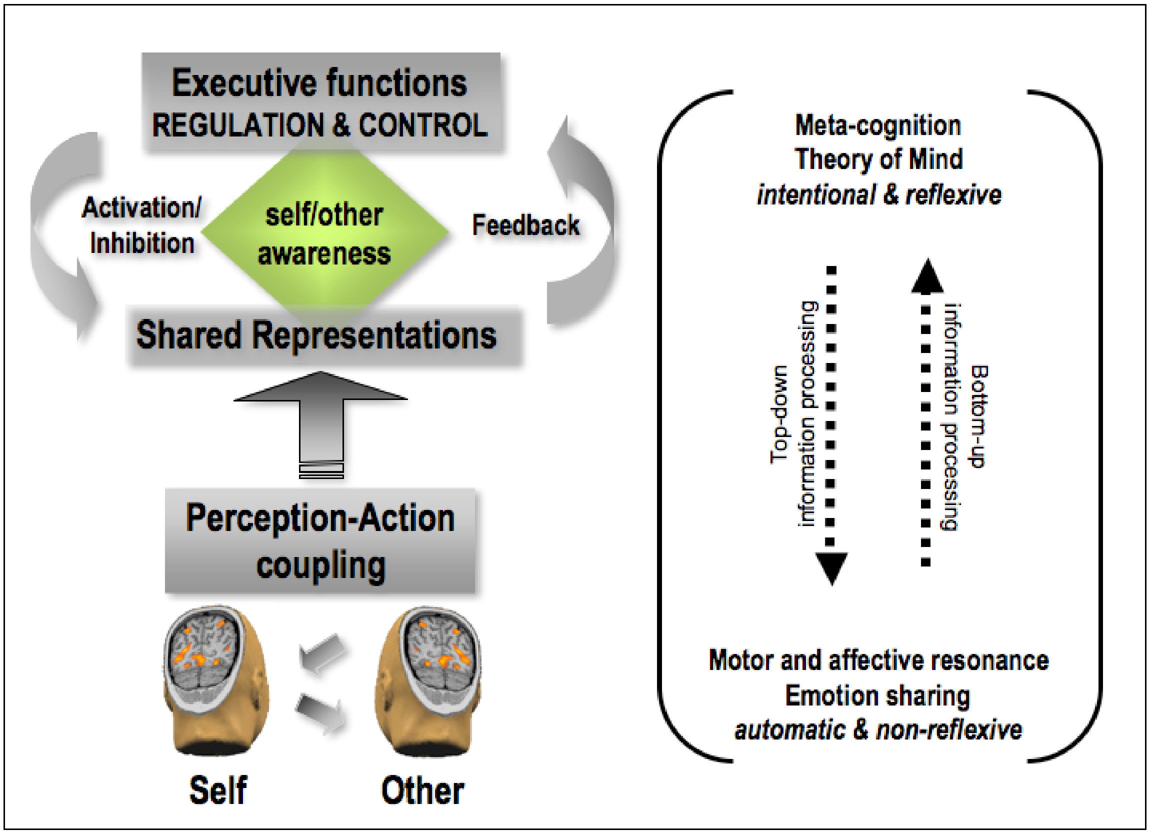

**FIGURE 1**. Schematic representation of the bottom-up (i.e., direct matching between perception and action) and top-down (i.e., regulation and control) information processes involved in human empathy. These two levels of processing are interrelated. The lower level, which is automatically activated (unless inhibited) by perceptual input, accounts for emotion sharing, which leads to the implicit recognition that others are like us. Executive functions, implemented in the prefrontal cortex, serve to regulate both cognition and emotion, notably through selective attention and self-regulation. This meta-level is continuously updated by bottom-up information and, in return, controls the lower level by providing top-down input. Thus, top-down regulation, through executive functions, modulates low levels and adds flexibility, making the individual less dependent on external cues. The meta-cognitive feedback plays a crucial role in taking into account one's own mental competence in order to react (or not) to the affective states of others.

impulse to care for offspring is almost certainly genetically hard wired. Less clear, however, is whether an impulse to care for siblings, more remote kin, and similar nonkin is genetically hard wired[10]. The emergence of altruism, of empathizing with and caring for those who are not kin, is not easily explained within the framework of neo-Darwinian theories of natural selection, and thus social learning explanations of kinship patterns in human helping behavior are highly plausible. Indeed, one of the most striking aspects of human empathy is that it can be felt for virtually any target, even targets of a different species.

Overall, this evolutionary conceptual view is compatible with the hypothesis that advanced levels of social cognition may have arisen as an emergent property of powerful executive functioning assisted by the representational properties of language[11]. However, these higher levels operate on previous levels of organization, and should not be seen as independent or conflicting with one another. Evolution has constructed layers of increasing complexity, from nonrepresentational to representational and meta-representational mechanisms, which need to be taken into account for a full understanding of human empathy. Representations, in this context, are defined as parallel distributed patterns of activation that reliably fire in response to a given stimulus. These neural networks encode information and, when temporarily activated, enable access to this stored information. This distributed nature of social processing poses a challenge to understanding the neuroscience of social cognition in general and empathy in particular. In addition, social psychological concepts, such as empathy, do not necessarily correspond to neuronal processes. Relations between psychological and biological processes cannot be understood fully by investigations at a single level of organization. Therefore, multilevel research is necessary to form bridges among disciplines and ultimately achieve a truly interdisciplinary social neuroscience[12].

## SHARED NEURAL CIRCUITS BETWEEN SELF AND OTHER

Prosocial behaviors may abound due to synchronizing representations between self and other. The initial component that precedes empathy draws on the somatic mimicry also known as emotion contagion, i.e., the tendency to automatically mimic and synchronize facial expressions, vocalizations, postures, and movements with those of another person, and consequently to converge emotionally[13]. Research demonstrates that viewing facial expressions triggers expressions on one’s own face, even in the absence of conscious recognition of the stimulus (e.g., [14]). It has been argued that initially such nonconscious behavioral mimicry may have had survival value by helping humans to communicate. Such a proposal is consistent with social psychology experiments showing that humans tend to nonconsciously mimic the behaviors of others[15], leading to more smooth interactions and increased liking. Interestingly, people with higher levels of dispositional empathy show this so-called chameleon effect to a greater extent. It has also been demonstrated that nonconscious behavioral mimicry increases affiliation, which serves to foster relationships with others[16].

Electrophysiological recordings in monkeys as well as neuroimaging experiments in humans have shown that the observation of actions performed by others activates cortical motor representations in the self (see [17] for review). The human mirror system is formed by a cortical network composed of the rostral part of the inferior parietal lobule, by the caudal sector (*pars opercularis*) of the inferior frontal gyrus, as well as by parts of the premotor cortex. Such an automatic perception-action mechanism has adaptive value for the survival of individuals. For instance, one fMRI study showed that observing fearful body expressions not only produces increased activity in brain areas associated with emotional processes, but also in areas linked with representation of action and movement[18]. Thus, the mechanism of fear contagion automatically prepares the brain for action.

Accumulating evidence suggests that similar neural circuits are stimulated when humans experience emotions and when they perceive others expressing emotions. For instance, the anterior insula gets activated in response to the sight of disgusted facial expressions of others as well as by the first-hand experience of disgust[19]. One fMRI experiment demonstrates that when participants are required to observe or to imitate facial expressions of various emotions, increased neurodynamic activity is detected in the superior temporal sulcus, the anterior insula, and the amygdala, as well as in areas of the premotor cortex corresponding to the representation of faces[20]. Another study showed that the observation of everyday hand and face actions performed with an emotion recruits regions involved in the perception and the experience of emotion and/or in communication[21]. The authors of that study speculate that, in addition to inducing resonance in the motor program necessary to execute an action, watching an action performed with emotion induces a resonance in the emotional system responsible for the affective modulation of the motor program. Such a mechanism could also be a key to understanding how the other person feels and to his or her associated intentions.

The action-perception coupling mechanism apparent in emotion contagion also seems to contribute to our ability to perceive and understand others' pain. Functional neuroimaging studies performed with healthy volunteers reveal that brain areas implicated in processing the affective and motivational aspects of pain mediate the observation of pain in others. In one study, participants in the scanner received either painful stimuli in some trials or, in other trials, observed a signal that their partner, who was present in the same room, would receive the same stimuli[22]. The first-hand experience of pain resulted in the activation of the somatosensory cortex, which encodes the sensory-discriminative dimension of a noxious stimulus such as its bodily location and intensity. During both types of trials, the anterior medial cingulate cortex (ACC), the anterior insula, and the cerebellum were activated. Interestingly, these regions contribute to the affective and motivational processing of noxious stimuli, i.e., aspects of pain that pertain to desires, urges, or impulses to avoid or terminate a painful experience. Similar results were also reported by Morrison and colleagues[23] who scanned participants during a condition of feeling a moderately painful pinprick stimulus to the fingertips and in another condition in which they witnessed another person's hand undergo similar stimulation. Both conditions resulted in common hemodynamic activity in a pain-related area in the right dorsal ACC. Common activities in response to noxious tactile and to visual stimulation were restricted to the right inferior Brodmann's area 24b. In contrast, the primary somatosensory cortex showed significant activations in response to noxious tactile, but not to visual, stimuli. The different response patterns in the two areas are consistent with the ACC's role in coding the motivational-affective dimension of pain, which is associated with the preparation of behavioral responses to aversive events. These findings are supported by an fMRI study[24] in which participants were shown still photographs depicting right hands and feet in painful or neutral everyday-life situations, and asked to imagine the level of pain that these situations would produce. Significant activations in regions involved in the affective aspect of pain processing, notably the ACC, the thalamus, and the anterior insula were detected, but no signal change in the somatosensory cortex. Moreover, the level of activation within the ACC was strongly correlated with subjects' ratings of pain attributed to the different situations.

Recently, a transcranial magnetic stimulation (TMS) study reported changes in corticospinal motor representations of hand muscles in individuals observing needles penetrating hands or feet of a human model[25], indicating that the observation of pain can involve sensorimotor representations. However, these findings are at odds with fMRI studies of empathy for pain, which did not detect any changes in the somatosensory cortex during the perception of pain in others[22,23,24].

One explanation for this discrepancy is that the TMS method senses subtle changes in the sensorimotor cortex that exist below the significance threshold in fMRI techniques. Another possibility is that attending to a specific body part elicits somatosensory activity in the corresponding region. This has been demonstrated in a positron emission tomography study in which participants were instructed to focus their attention either on the unpleasantness or the location of the noxious stimuli delivered on the participants' hands[26], with the latter condition resulting in increased regional cerebral blood flow in the contralateral primary somatosensory cortex.

Current evidence thus suggests that merely observing another individual in a painful situation yields pain-related responses in the neural network associated with the coding of the motivational-affective dimension of pain in oneself, and may include the somatosensory cortex as demonstrated by the TMS experiment. The finding of a strong overlap between first-hand experiences of pain and the perception of pain in others seems to suggest total congruence between self and other phenomenal experience. However, this assumption is superficial, as we do not literally feel the pain of others in such situations. Insights from anatomical studies related to pain experience and perception help to clarify this issue. Neuroimaging studies of first-hand experience and perception of pain in others indicate a partial overlap in the ACC and anterior insula, but also in specific nonoverlapping areas belonging to the pain matrix[27]. First-hand experience of pain is associated with more caudal activations (BA24), consistent with spinothalamic nociceptive projections, while perception of pain in others is represented in more rostral (and dorsal; BA32) regions, closer to the prefrontal pathways. A similar rostrocaudal organization is observed in the insula, in which pain-specific responses are observed in more medial-posterior parts, while observation of pain leads to increases in anterior insula[28]. This pattern of activation is also consistent with data collected using direct nonpainful and painful electrical stimulation of the insular cortex in neurological patients[29]. Painful sensations could only be evoked by stimulating the posterior part of the insula, while application of electrical currents to the anterior insula did not result in pain reports. Interestingly, the localizations of painful and nonpainful somaesthetic stimulations overlapped considerably, indicating a nonspecific role of the insula in the first-hand experience of pain.

It should also be noted that the neural network involved in the perception of pain in others (ACC and anterior insula) is not specific to pain processing. This network is also implicated in disgust and more generally in situations that can put individuals at risk, and which trigger visceral and somatosensory responses. Similarly, activation in the mid-ACC in conjunction with the pre-supplementary motor area (SMA) is not necessarily specific to the (either vicarious or first-hand) emotional experience of pain, but is related to other various processes, such as somatic monitoring, negative stimulus evaluation, and the selection of appropriate skeletomuscular movements of aversion[30]. Thus, it seems likely that the shared neural representations in the affective-motivational part of the pain matrix are not specific to the sensory qualities of pain, but are associated with more general survival mechanisms such as aversion and withdrawal.

## PERSPECTIVE TAKING AND EMPATHY

As noted above, human empathic abilities are more sophisticated than the simple yoking of perceptions of the self and the other. In the 18$^{th}$ century, Scottish philosopher and economist Adam Smith proposed that through imagination we place ourselves in the situation of another and enter as it were into his body, and become in some measure the same person. By means of imagination, we come to experience sensations, which are generally similar to, although typically weaker than, those of the other person. This capacity to engage in role taking has been theoretically linked to the development of empathy, moral reasoning, and more generally, prosocial behavior. Unlike the motor mimicry and emotional contagion aspect of empathy, perspective taking develops later, possibly because it heavily draws on the maturation of executive resources (i.e., the processes that serve to monitor and control thought and actions, including self-regulation, planning, cognitive flexibility, response inhibition, and resistance to interference) of the prefrontal cortex, which continues to mature from birth to adolescence. Interestingly, the development of self and other mental state understanding is functionally linked to that of executive functions[31,32]. There is increasingly clear evidence of a specific developmental link between theory-of-mind development and improved self-control at around the age of 4[33]. Carlson and Moses[34] have convincingly documented the contribution of executive functions, especially inhibitory control, in both the emergence and expression of mental state attribution in children. Perspective-taking ability allows us to overcome our usual egocentrism, tailor our behaviors to others' expectations, and thus make satisfying interpersonal relations possible[35]. In line with this reasoning, cognitive neuroscience research demonstrates that when individuals are asked to adopt the perspective of others, common neural circuits are activated both for the self and the other. However, taking the perspective of the other results in specific activation of parts of the frontal cortex that are implicated in executive control. It has been hypothesized that the role of the frontal lobes could be to hold separate perspectives, or to resist interference from one's own perspective[36].

In one functional neuroimaging study, the participants were presented with short written sentences that depicted real-life situations (e.g., someone opens the toilet door that you have forgotten to lock), which are likely to induce social emotions (e.g., shame, guilt, pride), or other situations that were emotionally neutral[37]. In one condition, participants were asked to imagine how they would feel if they were experiencing these situations, whereas in another condition, to imagine how their mother would feel in those situations. Reaction times were significantly longer when the subjects imagined emotional-laden situations as compared to neutral ones, both from their own perspective and from the perspective of their mothers. Neurodynamic changes were detected in the frontopolar cortex, the ventromedial prefrontal cortex, the medial prefrontal cortex, and the right inferior parietal lobule when the participants adopted the perspective of their mother, regardless of the affective content of the situations depicted. Cortical regions that are involved in emotional processing activated in the conditions that involved emotion-laden situations, including the amygdala and the temporal poles.

In one recent fMRI study, participants were shown pictures of people with their hands or feet in painful or nonpainful situations with the instruction to imagine themselves or to imagine another individual experiencing these situations[27]. Both the self-perspective and the other-perspective were associated with activation in the neural network involved in pain processing, including the parietal operculum, ACC, and the anterior insula. These results reveal the similarities in neural networks representing first- and third-person information, which is consistent with the shared representations account of social interaction[38]. However, the self-perspective yielded higher pain ratings and involved the pain matrix more extensively in the secondary somatosensory cortex, the posterior part of the ACC, and the middle insula. Thus, these results highlight important differences between the self- and other-perspectives. For instance, while the anterior insula and the ACC are activated both when participants imagine their own and when they imagine another's pain, the self-perspective is specifically associated with nonoverlapping clusters within the middle insula, another division of the ACC, and the right parietal cortex.

Of special interest are findings from social psychology that document the distinction between imagine the other and imagine oneself. These studies show that the former may evoke empathic concern (defined as an other-oriented response congruent with the perceived distress of the person in need), while the latter induces both empathic concern and personal distress (i.e., a self-oriented aversive emotional response such as anxiety or discomfort). This observation may help to explain why empathy, or sharing someone else's emotion, need not yield prosocial behavior. If perceiving another person in an emotionally or physically painful circumstance elicits personal distress, then the observer may tend not to attend fully to the other's experience and as a result lack sympathetic behaviors.

Combining a number of behavioral measures and event-related fMRI, a recent study by our group[39] investigated the distinction between empathic concern and personal distress using a set of ecological and extensively validated stimuli. This study further demonstrated that depending on the adopted perspective, observing other people in pain will trigger either empathic concern or personal distress, and that this other- vs. self-oriented response engages distinct neural networks. Participants were asked to watch a series of video clips featuring patients undergoing painful medical treatment either with the instruction to put themselves explicitly in the shoes of the patients ("imagine self"), or, in a second condition, to focus their attention on the feelings and reactions of the patients ("imagine other"). Behavioral measures confirmed previous social psychology findings that projecting oneself into an aversive situation leads to higher personal distress and lower empathic concern, while focusing on the emotional and behavioral reactions of another's plight is accompanied by higher empathic concern and lower personal distress (e.g., [40]). Neuroimaging data are consistent with this finding. The self-perspective evoked stronger hemodynamic responses in brain regions involved in coding the motivational-affective dimensions of pain, including bilateral insular cortices and anterior medial cingulate cortex (aMCC). In addition, the self-perspective was associated with stronger activation in the amygdala. This limbic structure plays a critical role in fear-related behaviors, such as the evaluation of actual or potential threats[41]. Interestingly, the amygdala receives nociceptive information from the spino-parabrachial pain system and the insula, and its activity appears closely tied to the context and level of aversiveness of the perceived stimuli[42]. Imagining oneself to be in a painful and potentially dangerous situation thus triggers a stronger fearful and/or aversive response than imagining someone else to be in the same situation. Alternatively and less specifically, the stronger involvement of the amygdala might also reflect a general increase of arousal evoked by imagining oneself to be in a painful situation. Regarding the insular activation, it is worth noting that it was located in the mid-dorsal section of this area (Fig. 2).

This part of the insula plays a role in coding the sensorimotor aspects of painful stimulation and it has strong connections with the basal ganglia[43], in which activity was also higher when adopting the self-perspective. Taken together, activity in this portion of the insula possibly reflects the simulation of the sensory aspects of the painful experience. Such a simulation might both lead to the mobilization of motor areas (including the SMA) in order to prepare defensive or withdrawal behaviors, and to interoceptive monitoring associated with autonomic changes evoked by this simulation process[44]. This finding gets additional support by another study of our group[27] in which activity in a similar part of the insula was also higher when participants adopted the self-perspective.

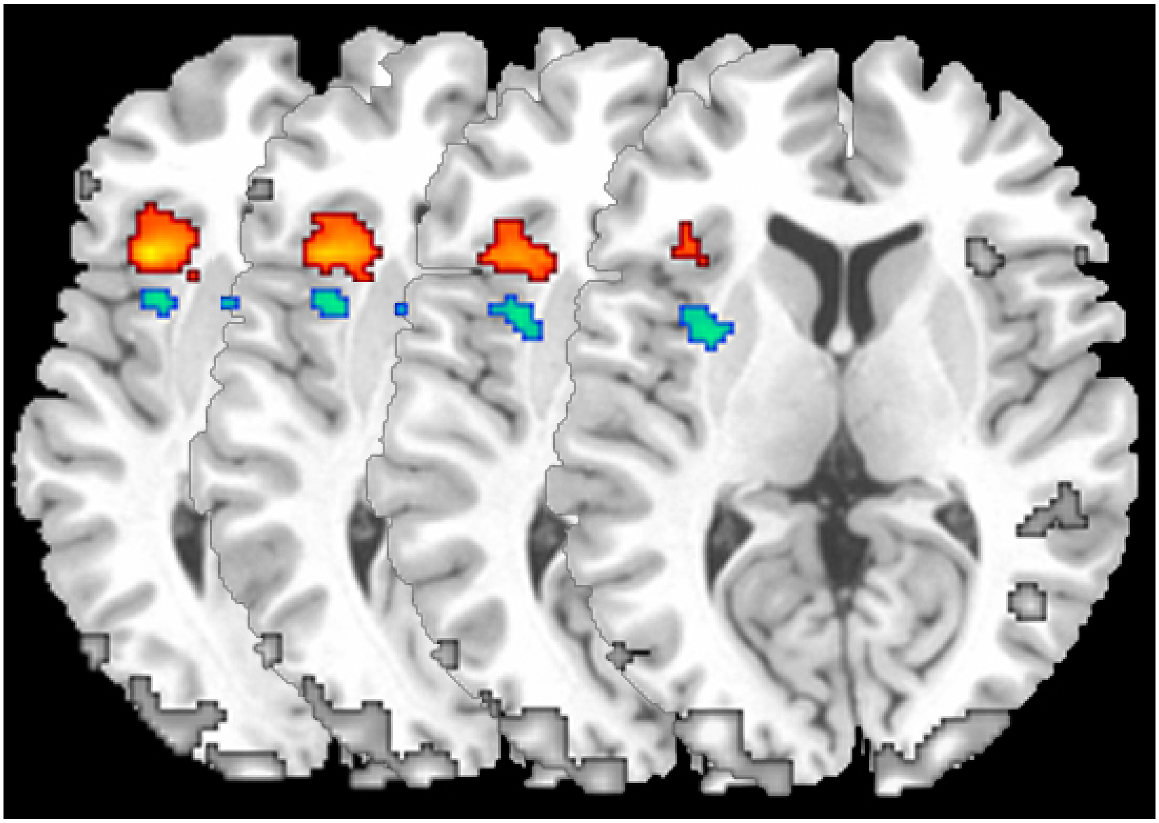

**FIGURE 2.** Dissociation of activation in the anterior and middle insular cortex elicited by perspective-taking instructions. Participants watched videos of patients undergoing painful medical treatment. The cluster in the anterior part of the insula (red to orange) shows activation irrespective of the perspective adopted by the study participants. The cluster in the mid-insula (blue to green) reflects higher signal change when participants adopted a first-person perspective as compared to adapting the perspective of the patient. It is worth noting that both perspectives activated the anterior insula, whereas the posterior cluster was specifically associated with the self-perspective (adapted from [39]).

Altogether, these empirical findings demonstrate the similarities in affective representations of self and other, stemming from shared neural circuits that can be activated by the act of perspective taking. These data also fit neatly with simulation theory, which states that behavior can be simulated by activating the same neural resources for acting and perceiving[45,46]. However, there are also some important differences in the activated neural systems involved in first- and third-person perspectives, which run counter to the suggestion of a complete merging between self and other. In the case of perception of pain in others, executive control may be necessary to inhibit distress experienced by the self and allow for the full, though nonegocentric and self-regulated, consideration of the other's situation.

## SELF-OTHER AWARENESS AND EMPATHY

Given commensurability between self and other action representations, whether these representations are activated through bottom-up processing (emotion sharing) or when adopting the subjective perspective of another individual, self-awareness and the sense of being an agent constitute key additional processes that are necessary for successful social interactions. The prerequisite for social communication, including the experience of empathy, is that the two agents can preserve their individuality. Social cognition relies both on similarities and differences between individuals. However, in the case of emotion sharing, a complete overlap between self and other representations would induce emotional distress (a self-oriented aversive emotional response), or empathic overarousal, which is not the goal of empathy[7]. In fact, in the experience of empathy, individuals must be able to disentangle their own feelings from the feelings shared with others to attribute mental states to the target. Self-awareness is a necessary condition for making inference about the mental states in others[47]. Therefore, “agency” is a crucial aspect for successfully navigating shared representations between self and other[48,49,50]. Further, agency plays a pivotal role in cognitive development, including the first stage of self-awareness (or pretheoretical experience of one’s own mentality), which scaffolds theory of mind capacities[51]. Indeed, the ability to recognize oneself as the agent of a behavior is the way one builds as an entity independent from the external world[52]. In the case of empathy, affective sharing must be modulated and monitored by the sense of whose feelings belong to whom[36], and thus, agency is a crucial aspect that enables a selfless regard for the other rather than a selfish desire to escape aversive arousal.

Accumulating evidence from neuroimaging studies, as well as lesion studies in neurological patients, indicates that the right inferior parietal cortex, at the junction with the posterior temporal cortex (temporoparietal junction, TPJ), plays a critical role in comparing signals arising from self-produced actions with signals from the environment[53,54]. The TPJ is a heteromodal association cortex that integrates input from the lateral and posterior thalamus, as well as visual, auditory, somaesthetic, and limbic areas. It has reciprocal connections to the prefrontal cortex and to the temporal lobes. Because of these anatomical characteristics, this region is a pivotal neural locus for self-processing that is involved in multisensory body-related information processing, as well as in the processing of phenomenological and cognitive aspects of the self[55]. Damage of this cortical area can produce a variety of disorders associated with body knowledge and self-awareness, such as anosognosia (i.e., denial of illness), asomatognosia (i.e., lack of awareness of the condition of all or parts of one’s own body), or somatoparaphrenia (i.e., delusional beliefs about the body[56]). For instance, Blanke and collaborators[57] demonstrated that out-of-body experiences (i.e., the experience that one’s self is located outside of one’s own body) can be induced by electrical stimulation of the TPJ in neurological patients.

A number of functional imaging studies have pointed out the involvement of the inferior parietal lobule in the experience of agency. Attribution of action to another agent, which crucially requires distinguishing between behavior of the self and the other, has been associated with specifically increased activity in the right inferior parietal lobe. In one study, experimenters used a device that allowed modifying the participant’s degree of control of the movements of a virtual hand presented on a screen. Experimental conditions varied the degree of distortion of the visual feedback provided to the participants about their own movements. Results demonstrated a graded hemodynamic activity of the right inferior parietal lobule that parallels the degree of mismatch between the executed movements and the visual reafference[58]. In another study[59], a joystick was used to drive a circle along a T-shaped path. Participants were informed that either they or the experimenter would drive the circle. In the former case, subjects were requested to drive the circle, to be aware that they drove the circle, and thus to mentally attribute the action seen on the screen to themselves. In the latter case, they were also requested to perform the task, but believed that the experimenter drove the action seen on the screen. Results showed that being aware of causing an action was associated with activation in the anterior insula, whereas being aware of not causing the action and attributing it to another person was associated with activation in the right inferior parietal cortex. In another fMRI study, participants were instructed to open and close their hand slowly and continuously (0.5 Hz) while this movement was filmed and projected to them online onto a screen[60]. A positive correlation between the extent of the temporal delay and activation in the right TPJ was found. Of particular interest, Uddin and colleagues[61] recently demonstrated selective impairment of self-other distinction when repetitive TMS was applied over the right inferior parietal lobule when participants performed a perceptual task involving discrimination between self-faces and other familiar faces. This latter study provides direct evidence for a causal role for this region in self-other discrimination.

In addition, studies on imitation have documented the involvement of right inferior parietal cortex/TPJ during reciprocal imitation in which it may be difficult to keep track of agency[62,63]. When participants imitated the other, the left TPJ was strongly engaged, whereas greater activation was detected in the right TPJ when they were being imitated. Only this latter condition involved discrepancies between predicted outcomes of the action performed by the participants and those perceived. These results provide strong arguments for the implication of the right TPJ in the process of agency by demonstrating a clear dissociation between left and right TPJ.

Interestingly, even mental simulation of one's own and others' behavior recruits a similar self-other discrimination mechanism. In one neuroimaging study, participants were requested to imagine familiar actions either from a first-person perspective or to imagine these actions from the perspective of another (known) person. Both perspectives were associated with signal increases in cortical regions involved in motor representations, including the premotor and left parietal lobule. Specific activation in the right inferior parietal cortex/TPJ was detected when participants mentally simulated actions from someone else's perspective[64]. Note that in both experimental conditions, participants were required to imagine an action. These results indicate that this area is not simply involved in associating actions and their sensory consequences, but, in general, contributes to distinguishing the self from others[38,65].

Furthermore, recent data suggest that this region is also involved in empathy[36] and in theory of mind, i.e. the awareness that others like the self possess mental states that can be similar or different from oneself[66,67]. For instance, the right TPJ is specifically involved when participants imagine how another person would feel in everyday-life situations that elicit social emotions[37] or in painful experiences, but not when they imagine these situations for themselves[27,39]. Such findings point to the similarity of the neural mechanisms that account for the correct attribution of actions, emotions, and thoughts to their respective agents when one mentally simulates actions or experiences emotions for oneself or for another individual. Consistent with this reasoning, a recent fMRI study of social perception and empathy demonstrated that activity in the inferior parietal cortex was negatively associated with the degree of overlap between self and other, and that less self-other overlap led to increased accuracy during social perception[68].

An alternative, though not contradictory, interpretation of the role of the right TPJ can be derived from studies on visual attention and the detection of uncertainty and of change. In a number of studies (see [69], for review), it has been demonstrated that the right TPJ is specifically activated when subjects focusing their attention on a certain screen location are presented with a stimulus at a nonattended location, representing a violation of their expectancy and requiring them to redirect their attention. Also, the work of Downar et al.[70] suggests that the right (and left) TPJs are crucial aspects of a multimodal cortical network for the detection of changes in the sensory environment. Thus, the higher activation in the TPJ region during studies on self-other discrimination and agency might reflect the higher uncertainty associated with the often unpredictable behaviors of our conspecifics. These data also suggest that elementary computational operations performed by the TPJ contribute to higher (social) levels of social interaction.

To sum up, self-awareness and the sense of agency play pivotal roles in empathy and significantly contribute to social interaction. These important aspects are likely to be at play in distinguishing emotional contagion, which heavily relies on the automatic link between perception of the emotions expressed by the other and one's own experience of the same emotion, and empathy that necessitates a more detached relation. We suggest that the nonoverlap in the neural response between self and other frees up processing capacity in the brain for unfolding appropriate future action towards the other. Further, being aware of one's own feelings, and being able to regulate consciously one's own emotions may be what allows us to distinguish empathic responses to others from our own personal distress, with only the former leading to prosocial behavior.

## EMOTION REGULATION

The capacity to regulate one's own emotions has a clear adaptive function for social interaction, both for the individual and the species. It has been demonstrated that individuals who can regulate their emotions are more likely to experience empathy, and also to act in morally desirable ways with others[71]. The construct of emotion regulation is defined as the process of initiating, avoiding, inhibiting, maintaining, or modulating the occurrence, form, intensity, or duration of internal feeling states, emotion-related physiological processes, emotion-related goals, and/or behavioral concomitants of emotion, generally in the service of accomplishing one's goal[72]. Emotion regulation is also important for modulation of one's own vicarious emotion so that it is not experienced as aversive. Previous research has shown that emotion regulation positively relates to feelings of concern for the other person[73]. In contrast, people who experience their emotions intensely, especially negative emotions, are prone to personal distress, i.e., an aversive emotional reaction, such as anxiety or discomfort based on the recognition of another's emotional state or condition[74]. Emotional distress may shift priorities toward the immediate present and therefore promotes a short-term focus; for instance, feeling better and getting relief from a painful situation, which may be in conflict with a motivation to feel for the other. In the case of empathy, the best response to other's distress may not be distress, but efforts to soothe that distress.

In our model of empathy, we consider that emotion regulation, under voluntary control (top-down processing), which taps into executive function resources, operates in conjunction with other neural mechanisms to carry out specific cognitive and emotional computations required for the experience of feelings for the other. Such computations include the selection of appropriate response and the inhibition of other less-appropriate responses.

Cognitive top-down mechanisms can either function to down-regulate (i.e., reduce) or up-regulate (i.e., promote) emotions. Although down-regulation seems to be more prevalent in the case of empathy, both processes play an important role when responding to others in need. In the case of perception of others in pain, the ability for down-regulation of emotion is prominent if the distress of the target becomes overwhelming for the observer. For example, a mother alarmed by her baby's cries has to cope with her own aversive reaction in order to provide appropriate care for her distressed offspring. Up-regulation, on the other hand, becomes important if an observer wants to promote his/her empathic response towards others, as in the attempt to relate to people to whom we would regularly react with indifference or even avoidance, such as members of an out-group. Various strategies have been identified to regulate emotions by means of top-down cognitive control[75]. Heuristically, these processes have been arranged on a continuum from attentional control to cognitive change[76]. While attentional control mainly affects the type and the amount of sensory information that is perceived and, hence, processed by the observer, cognitive change strategies predominantly tap into re-evaluating or reappraising external or internal affective information.

Research has shown that selectively focusing on specific sensory cues (such as facial expression or language prosody) conveying the emotional state of another may trigger different emotional responses in the observer. In line with this hypothesis, a recent fMRI study has shown that the amygdala is differentially involved when participants are instructed to attend to or to actively ignore fearful or happy faces as compared to houses[77]. Amygdala activation differed according to the valence of the facial expression and the category of the attended stimulus. For happy faces, activity in the amygdala was greater in the attend-face than in the attend-house condition, whereas for fearful faces, activity was greater in the attend-house than in the attend-face condition. Distraction is another powerful mechanism to exert attentional control, as it increases cognitive load and thus reduces the attentional resources available for stimulus perception and processing. In everyday situations, distractive strategies might play an important role if the cost of empathizing is too high, i.e., if we do not want pay attention to another's emotional state. Also, it is well known that distraction can effectively reduce and even eliminate the personal reaction to aversive painful stimulation[78]. A rostral region in medial prefrontal cortex (MPFC) seems to play an important role in this modulation[79], as activation in this region during painful stimulation was considerably reduced under higher attentional workload. In addition, the importance of the MPFC in regulating painful responses was recently corroborated by an fMRI study demonstrating that anxiety triggered by the anticipation of electric shocks is significantly reduced by the concurrent performance of a working memory task[80].

Further, selectively attending to certain aspects of a painful experience can increase (i.e., up-regulate) its aversiveness. This process recruits specific functional neural networks, as shown by a neuroimaging study[26]. In that study, focusing on the unpleasantness of noxious stimuli resulted in stronger activation in the medial pain system. This system includes the insula and medial cingulate cortices[81] and codes the affective and motivational dimensions of pain. In the case of empathy, the selective direction of attention might be one of the mechanisms yielding different emotional responses when using perspective-taking instructions. By means of top-down control, perspective-taking instructions force the participants to focus on different aspects of the emotional response of their conspecifics, or of themselves. As a result, participants' behavioral and neural responses are more self-centered when they adopt a first-person perspective. On the other hand, attending to the other in a more detached way results in an amplification of other-oriented responses.

Cognitive change strategies are an alternative and/or complementary way to regulate emotions. Denying the relevance of an emotion-eliciting stimulus is an example for such a strategy. Taking a detached observer position by consciously or unconsciously generating an image of the observing self is known to reduce the subjective experience of anxiety, sympathetic arousal, and pain reactivity. Such a detachment likely plays an important role in empathy in order to maintain a neutral perspective on the target; for example, in the interaction between a psychotherapist and his or her client. Recent fMRI studies have identified a limited number of regions in the anterolateral prefrontal and medial prefrontal cortices that mediate such function. For instance, participants instructed to imagine being in a safe, comfortable place showed reduced behavioral and physiological markers of anxiety related to the anticipation of painful stimulation[82]. This condition was accompanied by a modulation of activation in medial prefrontal/cingulate and anterolateral prefrontal cortex. While the former region seems to reflect the change in affective experience evoked by the emotion regulation strategy, activation changes in the latter are seen as their source, as this brain region has repeatedly been identified during emotion regulation by reappraisal.

Emotion reappraisal involves reinterpreting the valence of a stimulus in order to change the way in which we respond to it. It can either be intentionally achieved (e.g., in the attempt to react differently than usual to another's distress) or it can be based on additional external information provided about the emotion-eliciting stimulus. One recent study conducted by our group[39] investigated the effects of cognitive appraisal on the experience of empathy. In the MRI scanner, participants watched video clips from two groups of neurological patients expressing pain due to a new experimental treatment using dissonant sounds. Cognitive appraisal was manipulated by providing information about the effectiveness of that treatment: while health and quality of life improved in one group, members of the other group did not benefit from the treatment. Thus, stimuli of identically arousing and negatively valenced emotional content were watched with different possibilities to appraise the patients' painful responses. It was anticipated that witnessing another person suffering and knowing that his or her treatment had not been effective would increase the negative emotional response in the observer. Conversely, knowing that a treatment had been beneficial for the patient was expected to elicit down-regulation of the perceptually triggered affective response. In line with this hypothesis, distinct behavioral and neural responses were obtained in the study participants. Patients undergoing noneffective treatment were judged to experience higher levels of pain, and personal distress in the observers was generally more pronounced when watching videos of these patients. Brain activation was modulated in two subregions of the orbitofrontal cortex (OFC) and the rostral part of aMCC. The OFC plays an important role in the evaluation of positive and negative reinforcements[83], and is also involved in emotion reappraisal. For instance, attending to negatively valenced pictures evokes stronger activity in ventromedial OFC than reappraising these pictures in a way that they no longer elicit a negative response[84]. Activity in the OFC may thus reflect the requirement to evaluate the overall positive and negative aspects of the presented stimuli. Interestingly, watching effectively vs. noneffectively treated patients did neither modulate the hemodynamic activity in the visual-sensory areas nor in the insula. This suggests that both patient groups triggered an emotional reaction and that top-down mechanisms did not operate on perceptual processing at an early stage.

Overall, the capacity to regulate emotions is an important aspect of our ability to interact appropriately with other people. The prefrontal cortex is highly differentiated in terms of cell structures and patterns of interconnectivity with other cortical subsystems. In line with this fact, neuroimaging studies suggest that specific systems interact in generating emotion regulation. Social neuroscience is beginning to shed light on the physiological and neural mechanisms subserving the various emotion regulation strategies that allow us to better understand our conspecifics.

## MODULATION OF EMPATHY

Despite the fact that empathy partly relies on bottom-up information processing, which is automatic and unconscious, we do not always react to others empathetically in the conventional sense of the term. Mass communication and urban living conditions make us witness the distress and predicament of others on a daily basis, and yet we do not always respond in prosocial ways. This seems to be counterintuitive to lay conceptions that consider empathy as an automatic response that should be promoted without restrictions. However, despite the obvious advantages of understanding another person's thoughts and feelings, empathy does not come without costs[85]. Besides, empathy has evolved as an in-group phenomena[9]. Several intra- and interpersonal factors have been identified that facilitate or inhibit the occurrence and the degree of empathic responses, and thus allow modulation of their associated expenses.

Social neuroscience currently investigates the neural mechanisms underlying such modulations. Background affective state, prior experience with a situation, and the ability to cope with the distress of others (which relies, among others, on emotion regulation capabilities) are just a few examples of various intrapersonal factors that can impact the experience of empathy. It has been shown that experimentally induced emotional states affect the ability of observers to recognize the facial display of emotion[86]. Depressive mood is also known to affect our perception of the world, including the expression of emotions by others. A recent developmental neuroscience study identified the potential neural substrates of such a modulation. Limbic structures such as the amygdala and the nucleus accumbens became hyperactive when participants with pediatric bipolar disorder attended to the facial expression of emotion. Similarly, patients with generalized social phobia, which are characterized by fear of social interactions and sensitivity to disapproval by others, show increased amygdala activation when exposed to angry or contemptuous faces[87]. The effects of affective state do not only become prevalent in psychiatric or psychological disorders, nor are they confined to the evaluation of emotional information. Indeed, emotional priming (i.e., the brief presentation of negatively or positively valenced stimuli before presenting a target stimulus) enhances early visual processing in normal volunteers[88]. This, in turn, might affect the visual perception of emotional cues, such as the subtle changes in the facial expression of emotion. Prior experience is another factor that affects our emotional resonance with others. For example, promoting knowledge about the specific needs and problems of elderly people in a game situation resulted in significant increases in empathy and caring for elderly patients[89]. This game included exercises such as wearing a pair of heavy rubber gloves to simulate decreased manual dexterity, and goggles with a film of clear tape over the lens to simulate cataracts

Whether dispositional empathy, i.e., the self-reported general tendency to respond empathically to others, also modulates the occurrence and intensity of empathic responding is currently a matter of debate. Part of this controversy is related to the notoriously low validity of self-report measures in predicting actual empathic behavior[90]. Nevertheless, several recent neuroimaging studies demonstrate specific relationships between questionnaire measures of empathy and brain activity. For example, Lamm and colleagues[39] demonstrated significantly increased activation in insular and cingulate cortices in participants with higher self-reported empathy during the observation of others in pain. This is in line with findings by Singer and colleagues[22,91], and shows modulation of activity in the very brain regions that are involved in coding the affective response to another's distress. Note, however, that no such correlations were found in a similar study[24].

Cognitive and affective dispositions towards the other, or the type of relationship and the associated attachment (e.g., parents and their offspring), also affect how we respond to others. These interpersonal factors do not necessarily occur in isolation with the intrapersonal factors, but are likely to interact with them. This is demonstrated by an fMRI study showing significant interactions between gender and parental status in the neural response to infant crying and laughing[92]. Women, but not men, and independent of their parenthood, showed neural deactivation in the anterior cingulate cortex in response to infant crying and laughing. In addition, the response pattern in the amygdala and interconnected limbic structures changed fundamentally with parental experience in both genders. Nonparents showed stronger activation from laughing, whereas parents showed stronger activation for crying. These results indicate that the emotion-sharing component may be subject to personal experience, and/or that emotion regulation and associated caring is prepared differently in men and women (but see also [93]). Additional evidence for this effect was provided by an fMRI study that demonstrated that mothers viewing their offspring vs. a familiar child showed stronger responses in a (para)limbic brain network and in posterior superior temporal sulcus (STS), possibly reflecting the more intense attachment that mothers have to their own offspring.

The question “how do competitive vs. cooperative interpersonal relationships affect empathy?” has been investigated both at the behavioral, psychophysiological, and neural levels. The reasoning behind these studies is that affective dispositions, or attitudes, differ depending on whether the other is seen as a competitor or a cooperator, and in turn influence whether we react with a congruent or a noncongruent emotion to another’s affect. Lanzetta and coworkers[94,95] investigated the psychophysiological, behavioral, and psychological effects of attitudes on interpersonal interaction. Their main finding is that competitive relationships lead to asymmetric affective (counterempathic) responses, while cooperative settings result in symmetric vicarious emotions. For instance, psychophysiological measures indicated that participants reacted to a painful shock of competitors with little arousal and distress, but got distressed when seeing them in joy. The reverse pattern was obtained for cooperators. These findings reflect an important and often ignored aspect of empathy, namely that this ability can also be used in a malevolent way as when knowledge about the emotional or cognitive state of competitors is used to harm them (e.g., when enemy soldiers are attacked after exhausting them by continuous artillery barrage). A recent study revealed the neural correlates of such counterempathic responding. Singer and colleagues[91] engaged participants in a sequential Prisoner’s dilemma game with confederate targets, who would either play the game in a fair or unfair way. Following this behavioral manipulation, fMRI measures were taken during the observation of fair and unfair players receiving painful stimulation. Observing fair players replicated earlier findings of increased activation in brain areas coding the affective aspect of pain, such as the anterior insula and medial/anterior cingulate cortex. Of particular interest, activation in these brain regions was significantly reduced when participants in the scanner observed unfair players in pain. This effect, however, was detected in male participants only, who also showed a concurrent increase of activation in reward-related areas (i.e., nucleus accumbens and ventral striatum). The specific reduction of activity in the neural network processing the affective dimension of pain is in contrast with the results from the neuroimaging studies that investigated the modulation of affective responses (vicarious or direct) to pain by means of emotion regulation (e.g., [39,82]). Indeed, these studies reported modulation of activity in brain regions that are not directly involved in representing the affective state, such as the orbitofrontal cortex (which, interestingly, was also activated in male participants in Singer’s study) or the medial prefrontal cortex. This suggests that different mechanisms are at play when empathy is modulated due to inter- or intrapersonal factors. Liking/disliking somebody might determine whether an affective empathic response is generated at all, while responding to the distress of neutral others might require regulation of a readily evoked emotional response. Alternatively, differences in the paradigms used (display of dynamic emotional expressions vs. cues for painful stimulation) might explain these differences between studies.

In sum, there is strong behavioral evidence demonstrating that the experience of empathy can be modulated by a number of social-cognitive factors. In addition, a few recent neuroscience studies indicate that such a modulation leads to neurodynamic activity changes in the neural systems that process social information. Further studies are required to increase our knowledge about the various factors, processes, and (neural and behavioral) effects involved in and resulting from the modulation of empathic responses.

This knowledge will inform us how empathy can be promoted to ultimately increase humankind's ability to act in more prosocial and altruistic ways.

## CONCLUSION

The construct of empathy has been discussed in philosophy of mind for many decades and extensively investigated by social psychologists as well as by developmental psychologists. More recently, cognitive neuroscience has begun to unravel the neural systems that underpin the processes involved in the experience of empathy, including emotion sharing, perspective taking, and emotion regulation. The new approach of social neuroscience, by combining research designs and behavioral measures used in social psychology with neuroscience markers, can play an important role in disambiguating competing theories in social psychology in general and in empathy-related research in particular. For instance, one critical question debated among social psychologists is whether perspective-taking instructions induce empathic concern and/or personal distress, and to what extent prosocial motivation springs from self-other overlap (see [7,96]). The recent work reviewed here demonstrates that adopting a self-perspective when observing others in pain results in stronger feelings of personal distress and activates the pain matrix to a larger extent, as well as the amygdala. Such a complete self-other merging seems to be detrimental to empathic concern. Indeed, the best response to another person's plight may not be distress, but efforts to soothe that distress. Conversely, when participants take the other's perspective, there is less overlap between the neural circuits involved in the processing of first-hand experience of pain, and they indeed report more feelings of empathic concern. From these studies, it can be concluded that empathy relies both on bottom-up information processing (shared neural systems between first-hand emotional experience and the perception or imagination of the other's experience), as well as top-down information processing that allows modulation and self-regulation. Without self-regulation, information processing would lose flexibility and would become primarily bound to external stimulations.

Finally, our paper illustrates the relevance of combining neuroscience research with social psychology in the validation of social science models of social interaction. The convergence of approaches also has benefits the other way around, in the sense that paradigms and models from social psychology play a complementing (and inspiring) role in the investigation of the brain mechanisms underpinning social behavior (see [97]). It is our conviction that a more comprehensive understanding of empathy and related emotions will be fostered by integrative analyses that span the biological and social levels of organization. Such a perspective also has the potential for generating new hypotheses concerning social cognitive disorders.
